# Role of micro-RNAs in drug resistance of multiple myeloma

**DOI:** 10.18632/oncotarget.11032

**Published:** 2016-08-02

**Authors:** Jahangir Abdi, Hou Jian, Hong Chang

**Affiliations:** ^1^ Division of Molecular and Cellular Biology, Toronto General Research Institute, Toronto, Ontario, Canada; ^2^ Department of Laboratory Medicine & Pathobiology, University of Toronto, Toronto, Ontario, Canada; ^3^ Department of Hematology, Shanghai Chang Zheng Hospital, Shanghai, China; ^4^ Department of Laboratory Hematology and Medical Oncology, University Health Network, Toronto, Ontario, Canada

**Keywords:** miRNA, drug resistance, multiple myeloma

## Abstract

While novel therapeutic approaches have profoundly improved survival of multiple myeloma (MM) patients, drug resistance and treatment refractoriness still persists. This obstacle highly demands thorough investigation into the root and underlying molecular mechanisms to develop more effective strategies. The advent of micro-RNAs (miRNAs) in the study of cancer biology and pathogenesis in recent years has revolutionized therapy in this field and particularly opened new windows to further understanding of tumor drug resistance. However; in spite of the fact that miRNAs involvement in MM pathogenesis and progression has been substantially evidenced, miRNA investigation in MM drug resistance is still in its infancy. Our knowledge of the potential role of miRNAs in MM drug resistance comes from few recent reports confirming that some miRNAs including miR-137/197, miR-21 and miR-221/222 could negatively modulate drug sensitivity of MM cells. Further continuous researches are required to exploit miRNAs to elucidate the critical mechanisms controlling drug resistance in MM. In this review, we will highlight the most recent observations on the role of miRNAs in MM drug resistance. Moreover, approaches and insights into clinical application of miRNAs to overcome MM drug resistance will be discussed.

## INTRODUCTION

In the era of advanced technologies in cellular and molecular genetics, our understanding of the clonal root, pathogenesis and progression of multiple myeloma (MM) is remarkable. Efficient techniques and tools have helped diagnosis of MM and its pre-malignant stages with noticeable certainty. During the past decades and most recent years, enormous studies have been trying to identify the mechanisms of MM disease development, tumor expansion, biologic and clinical response to drugs and perhaps more diligently MM drug resistance. Indeed, development of drug refractoriness and relapse is the most critical and as-yet not overcome obstacle in MM treatment hampering most recent and novel therapies and imposing significant patient-related economic costs. To tackle this issue, more and more in depth studies are demanded to probe the complex mechanisms underlying drug resistance in MM to develop more powerful therapeutic agents.

microRNAs (miRNAs) tend to increasingly attract attentions as the important gene regulatory elements in the study of pathobiology of many cancers including MM [1, 2]. These small non-coding RNA molecules play substantial roles in post-transcriptional negative regulation of a multitude of human genes including oncogenes and tumor suppressors. miRNAs effectuate these functions through partial complimentary binding of their seed sequences (2∼7nt long) to 3′UTR sites in target mRNAs leading to translational repression or mRNA degradation [3]. miRNAs can function as oncomiRs if their targets are tumor suppressors (e.g. TP53, PTEN) or as tumor suppressors if their targets are oncogenes (e.g. MYC, MDM2) [4]. A growing body of evidence supports the role of both categories in regulation of drug response of various human cancers [5, 6], however; our knowledge of how these players could really be involved in drug resistance of MM is limited. Nonetheless; the findings of a few recent studies suggest that some miRNAs may regulate drug response of MM cells through modulation of apoptotic, anti-apoptotic or proliferative pathways [7-10]. Regarding the facts that MM drug resistance is highly complicated and also each miRNA can target at least 200 genes [11]; research on the roles of miRNAs in MM drug resistance and whether they would be utilized as therapeutic targets to overcome this resistance still have a long way to go.

In this review, we will discuss the concept of miRNA involvement in drug resistance in MM by putting forward the latest relevant findings and also some inference from observations in other cancers.

## MIRNA INVOLVEMENT IN DRUG RESPONSE OF MALIGNANT CELLS: GENERAL CONCEPT

**Figure 1:**
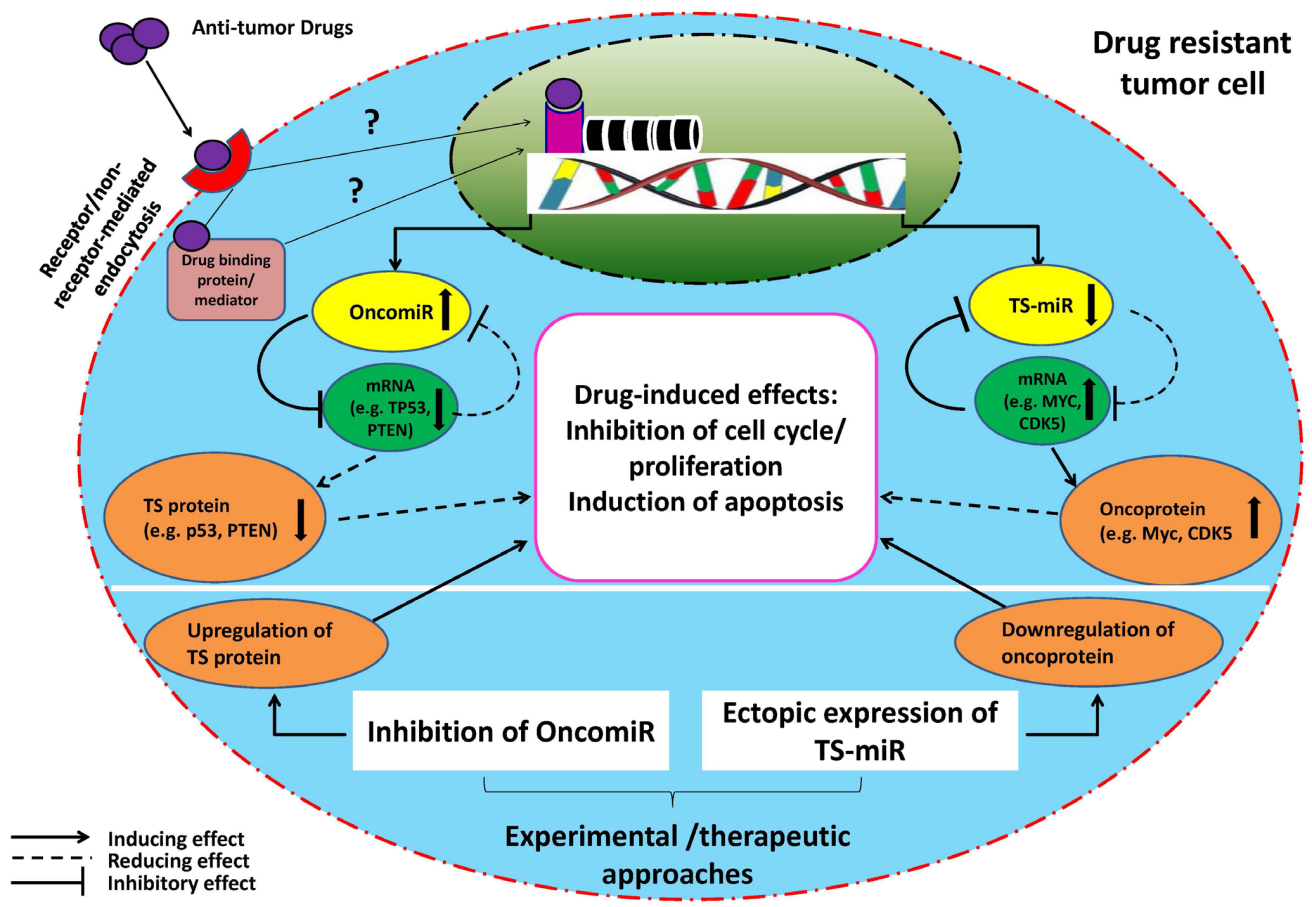
Schematic illustration of proposed miR-(miR's) target-drug network in a drug resistant tumor cell The model indicates that the function of a drug (an outsider) will be compromised by an endogenous miRNA-target axis in a tumor cell resistant to anti-tumor drug. Moreover, it suggests that endogenous low (TS-miR) or high (OncomiR) miRNA expression could help maintain a drug resistant phenotype. In such a context, OncomiRs interact with and suppress their targets (basically pro-apoptotic or tumor suppressor genes) leading to decrease in drug-induced effects (such as apoptosis, inhibition of cell cycle/proliferation). On the contrary, underexpressed TS-miRs interact with their targets (mostly anti-apoptotic genes or oncogenes) but fail to suppress them efficiently resulting in the same outcomes as above. This established mutual interaction between above miRNAs and their targets is usually understood through experimental gain- or loss-of-function studies on miRNAs where drug resistant tumor cells will become sensitive to the drugs. However; it is not clear whether miRNA alterations in drug resistant tumor cells occur due to gain or loss of genetic material during drug exposure (and whether the drug affects genome directly or indirectly [88, 89]). TS = tumor suppressor

In recent years many studies have documented the role of miRNAs in growth, proliferation and survival of cancer cells (reviewed at [12]), and interesting findings pinpoint interaction of miRNA networks with oncogenic pathways [13]. Whole genome investigations have disclosed dysregulation of miRNAs in human malignancies and have provided evidence that miRNAs in fact play dual roles in cancers: miRNAs can modulate oncogenic or tumor suppressor pathways including p53, c-MYC, RAS, on the other hand expression of miRNAs themselves can be regulated by oncogenes or tumor suppressors [14]. Indeed, the best known oncogenes and tumor suppressor genes are powerful regulators of cell growth, proliferation and apoptosis [15, 16]. Irrespective of whether miRNAs' targets are tumor suppressors or oncogenes, their specific functional effects will be compromised in the neoplastic context and in either case miRNAs effects may be exploited in favour of tumor conditions optimization. For instance, the oncogene MDR1 is a known target of miR-451 which is downregulated in MCF-7/DOX cell lines [17]. Low expression of this miRNA will relieve the suppressive effect of miR-451 on above cells hence upregulation of MDR1 and induction of drug resistance. On the contrary, for an oncomiR like miR-125a-5p which is overexpressed in several cancers [18], the tumor suppressor target (e.g. p53) will be downregulated culminating in the same outcomes as mentioned. In both cases, cancer cells could gain some level of resistance when exposed to chemotherapeutic drugs. However; in an experimental setting ectopic expression of a tumor suppressor miRNA or knock-down of an oncomiR should lead to, respectively, downregulation and upregulation of their targets resulting in tumor cell apoptosis or enhancement of drug-induced apoptosis (see also Figure 1). These findings pinpoint the existence of an established functional feedback loop between miRNAs and their potential targets controlling drug response of cancer cells. Indeed the concept of miRNA link with drug resistance/sensitivity of cancer cells emerged due to the fact that miRNAs could also regulate cell death [19] making these small non-coding RNA molecules interesting therapeutic targets in cancer treatment.

## INVOLVEMENT OF MIRNA ABNORMA-LITIES IN MYELOMA PATHOGENESIS

During the past decade and also in recent years, enormous studies have yielded reliable wealth of evidence supporting the role of miRNAs in MM pathogenesis, prognosis and clinical outcomes. The first evidence of miRNA involvement in MM pathogenesis was presented in 2005 by Al Masri et al. who showed that MM cell lines and MM patient samples displayed a lower expression of miR-125b, miR-133a, miR-1, miR-124a, miR-15 and miR-16 than their normal counterparts [20]. Distinct expression pattern of several miRNAs between MGUS and overt MM samples compared with normal controls also suggests role of some miRNAs in MM progression [21]. For example, miR-21 was upregulated in both MGUS and MM, but miR-17-92 cluster was upregulated only in MM. Lionetti et al. found let-7e, miR-125a-5p, and miR-99b highly upregulated in t(4;14) patients, and some miRNAs associated with allelic imbalances or loss of heterozygosity sites including let-7b at 22q13.31 or miR-140-3p at 16q2 [22]. Several other studies have also shown the associations of miRNAs with MM high risk groups, survival and isotypes [23, 24]. Most of these studies attempted to exploit miRNA expression patterns to discriminate MM, MGUS and normal donors and also characterize associations with different molecular subtypes/subgroups. However; whether miRNA expression pattern is really altered in MM patients who are under drug treatment, refractory to treatment or at relapse hence possible involvement in drug resistance has not been clearly explored. Nonetheless, implication for role of miRNAs in resistance to bortezomib (BTZ) in MM was provided by Neri et al. in their study presented in a meeting in 2009 [25]. They found a different miRNA signature in BTZ resistant cell lines compared to BTZ sensitive lines and, interestingly, miRNAs modulated in MM cells isolated from BTZ-resistant MM patients clustered with those in BTZ-resistant cell lines, however; further mechanistic approaches were not taken by the authors. In a quite recent publication, Zhang et al. [26] showed that exosomal miRNAs, miR-16-5p, miR-15a-5p, miR-20a-5p and miR-17-5p, in circulating exosomes were significantly downregulated in BTZ-resistant MM patients suggesting correlation of these miRNAs with BTZ resistance. The study did not follow further functional or mechanistic explorations.

## MIRNA-TARGET NETWORKS AND DRUG RESISTANCE IN MM

Identification of miRNAs involved in modulation of the effects of drugs is becoming an attractive research focus in cancers [5, 27], although still in its infancy in MM. In fact, the recent amazing findings that miRNAs are able to determine the efficacy of chemotherapeutic drugs have given rise to the field of “miRNA pharmacogenomics” [27]. In these contexts, miRNAs are basically parts of a triad consisting of miRNA-target-drug which function to reduce the effect of the drug probably through modulation of miRNA, the target gene or both. Perhaps the best example of this scenario is resistance to 5-fluorouracil induced by miR-21 through targeting of the tumor suppressor PTEN [28]. On the other hand, Ballabio et al. [29] observed that endogenous level of miR-27a was low in BTZ-resistant MM cell lines and ectopic expression of miR-27a in these cells sensitized them to BTZ through suppressing CDK5 which is an oncogene with high expression and associated with lower survival in MM [30]. CDK5 has also been shown to modulate BTZ sensitivity of MM cells [31]. The general concept of miRNA-target-drug network is illustrated in Figure 1.

### miRNAs involvement in drug response of MM cells

To gain an idea of miRNA involvement in MM drug resistance, several experimental approaches have to be taken:

1-miRNA analysis in some sets of human myeloma cell lines (HMCLs) including drug resistant and parental lines and also primary CD138+ MM cells from bone marrow of MM patients at relapse or refractory to treatment and normal donors. In an attempt, Munker et al. [32] profiled miRNA expression in two HMCLs, RPMI8226 and U266 and their resistant variants RPMI8226/Dox6 and RPMI8226/LR5, U266Dox and U266/LR7, respectively. They found that in comparison of individual resistant cell lines with their respective parental lines, multiple miRNAs were up- or downregulated. However, they verified the chip data of only 3 miRNAs, miR-21, miR-181a/181b and miR-565 with RT-PCR and concluded that only miR-21 was commonly upregulated in all melphalan-resistant clones relative to the parental ones. Their investigation will raise the question as to whether miRNA alterations were really due to an oncogenic transformation in the first place or appeared solely because of loss or gain of genetic material in the course of establishment of drug resistance. Their observations would have been more supported if they had used primary MM cells from drug resistant patients to compare with those from normal donors. Notably, miR-21 has been shown to function as a potential oncomiR in a variety of cancers [28, 33-35] including MM. One study demonstrated that miR-21 could induce resistance to apoptosis triggered by dexamethasone, doxorubicin, or BTZ in MM cells [8]. The same group also showed that adhesion to bone marrow stromal cells (BMSCs) upregulated mi-21 in MM cells and induced drug resistance by targeting RhoB, also inhibition of miR-21 inhibited MM cells proliferation and induced apoptotic cell death. A recent study by Leone et al. provided further evidence that the oncogenic miR-21 was indeed a potential therapeutic target in MM, although they did not explicitly addressed the question whether miR-21 controls drug response of MM cells [36]. Another important oncomiR is miR-221/222 cluster on chromosome X which is reported to be overexpressed in various malignancies including MM [7, 9, 10, 37, 38]. Zhao et al. [9] using MM1R and MM1S cell lines as models of dexamethasone resistance and sensitivity, respectively, showed higher expression of miR-221/222 cluster in MM1R cells than in MM1S cells.

2-Analysis of functional response of drug resistant HMCLs or primary MM cells from MM patients (at relapse or refractory to the drugs) following ectopic expression of “downregulated miRNAs” or inhibition of “up-regulated miRNAs” and comparison with normal counterparts in all settings. In a recently published work [39], our group found two tumor suppressor miRNAs, miR-137 and miR-197, downregulated in HMCLs (MM1S, MM1R, My5, H929 and 8226) and MM patients' tumor cells compared to normal counterparts. However; H929 and U266 cell lines had higher base line levels of these miRNAs. Further inhibition of these miRNAs using synthetic inhibitor oligos in H929 cells, increased viability of the cells and rendered them a bit resistant to BTZ. On the contrary, overexpression of the two miRNAs in U266 or H929 cells reduced cell viability. These findings implicate a role of miR-137/197 in drug resistance of MM cells. Further exploration into the potential mechanisms controlling these effects is still ongoing. Zhao et al. [9] demonstrated that miR-221/222 inhibition partially reversed dexamethasone sensitivity of MM1R cells while miR-221/222 treatment made MM1S cells more resistant to dexamethasone. Their gain and loss of function studies also confirmed PUMA as the direct target of miR-221/222. Interestingly, they found high expression of miR-221/222 and low expression of PUMA in MM cells isolated from MM patients at relapse compared with normal controls suggesting that inhibiting miR-221/222 would be a promising therapeutic target in MM. In line with this study, another group also showed that miR-221/222 expression was higher in melphalan-resistant HMCLs compared with parental lines, inhibition of miR-221/222 induced apoptosis, induced expression of PUMA and overcame melphalan resistance even in the presence of BMSCs [10]. They also found that inhibition of miR-221/222 modulated drug influx-efflux transporters SLC7A5/LAT1 and the ABC transporter ABCC1/MRP1, further supporting the role of these miRNAs in possibly multiple drug resistance in MM cells.

### Mechanisms of miRNA-mediated drug resistance in MM cells

As mentioned above, miRNAs function through their targets which act like switches relaying miRNAs to modulation of multiple cellular functions. According to the literature, 3 signaling pathways mostly known to be dysregulated in cancers, NFκB, Ras and p53, have been found connected with function of various miRNAs [40]. In addition, genome-wide networks of miRNAs have been found associated with other important oncogenic pathways such as Myc, IFN and STAT [13]. Although the vast majority of miRNA studies in MM have been focused on clinical / subgroup correlations and their role in cell viability and proliferation, only few studies addressed miRNA and drug resistance specifically in MM drug resistant models. In a few recent studies in MM, mostly p53-related signaling pathway has been found modulated by miRNAs in the context of drugs suggesting a possible mechanism of miRNAs in regulation of drug response in MM cells (Table 1).

**Table 1:** miRNAs studied with potential role in MM drug resistance

miRNA studied	Observed alteration	Targets identified	Functional responses	Refs
21	Up-regulated in melphalan-resistant HMCLs	NA	NA	[36]
21	Up-regulated in MM cells following adhesion to BMSCs	RhoB	Enforced expression of miR-21 led to reduced apoptosis induced by DEX, DOX and BTZ. Inhibition of this miRNA left the opposite effects	[8]
27a	Downregulated in BTZ-resistant HMCLs	CDK5	Ectopic expression of miR-27a in MM cells increased their sensitivity to BTZ	[25,29]
221/222	Upregulation in melphalan-resistant HMCLs	PUMA/BBC3	miR-221/222 inhibitor upregulated PUMA increasing apoptosis in drug resistant HMCLs. This treatment also suppressed ABC transporter ABCC1/MRP1 and upregulated L-type amino acid transporter SLC7A5/LAT1	[7,10]
221/222	Upregulated in DEX-resistant HMCL (MM.1R)	PUMA/BBC3	Inhibition of miR-221/222 in MM.1R cells partially restored their DEX sensitivity, whereas enforced expression in MM.1S cells downregulated PUMA and rendered them resistant to DEX	[9]
125a	Upregulated in MM cells following adhesion to BMSCs	p53	NA	[41]
125b	Upregulated in DEX-responsive MM cells	p53, interacts with miR-34a targeting SIRT1 histone deacetylase	Anti-miR-125b increased p53, miR-34a, decreased SIRT1 and increased DEX-induced apoptosis	[42]
137/197	Suppressed in MM cells harboring 1p12-21 deletion	MCL-1	Further inhibition in H929 cells induced resistance to BTZ	[39]
451	Up-regulated in MM side population (MM cancer stem cells), an implication of this miRNA in MM drug resistance	tuberous sclerosis 1 (TSC1)	Anti-miR-451 enhanced apoptosis induced by BTZ, As2O3 and melphala, decreased clonogenicity, and reduced MDR1 mRNA expression in SP cells	[43]

Leotta et al. [41] found miR-125a-5p upregulated in MM cell lines and patients' tumor cells compared to normal controls. By transfecting MM cells with miR-125a-5p mimics, expression of p53-related genes declined, whereas inhibition of miR-125a-5p reduced cell growth and migration, and increased apoptosis. Interestingly, they also observed that BMSCs further upregulated miR-125a-5p in MM cells and in fact modulated the miR-125a-5p/p53 axis. Although modulation of drug response by miR-125a-5p was not a goal in this study, it might be conceived from their observations that miR-125a-5p could at least play role in BMSC-induced drug resistance.

In addition, miR-125b has been shown to induce dexamethasone resistance in MM cells by upregulating miR-34a and suppressing SIRT1 deacetylase thus allowing maintained acetylation and inactivation of p53 through the p53/miR-34a/SIRT1 signaling network [42]. Silencing of miR-125b upregulated p53 and enhanced dexamethasone-induced apoptosis. miR-125b was also reported to function as an oncomiR to mediate BTZ resistance of cutaneous T-cell lymphoma (CTCL) cells by targeting tumor suppressor MAD4 [44] and in pediatric acute lymphoblastic leukemia (ALL) cells to induce resistance to ATRA and doxorubicin by targeting tumor suppressor Bak1 [45] further supporting the notion that miR-125 family contribute to drug resistance as oncomiRs. However; these observations are not consistent with what obtained by another group, who showed that miR-125b was in fact suppressed in MM cells and its ectopic expression triggered apoptotic and autophagic MM cell death [46].

In the aforementioned studies by Gulla et al. and Zhao et al. [9, 10] PUMA, a (BH3)-only Bcl-2 family member, and a critical mediator of p53-dependent and -independent apoptosis, was identified as the direct target of miR-221/222 cluster. BAX and BAK, two other p53-related genes, were also upregulated following inhibition of miR-221/222 *in vitro* and in retrieved tumor samples from xenograft models. Their experiments showed that direct targeting of the miR-221-222 cluster could abrogate this general mechanism of drug resistance. They suggest that as PUMA is a downstream regulator of apoptosis, it is likely that anti-sense-miR-221/222 therapy should not only overcome dexamethasone resistance but also resistance to other drugs, including Lenolinamide, as well as p-53-dependent and -independent mechanisms of drug resistance. Interestingly, a recent study demonstrated that miR-221 expression was regulated by RelB-p52 complex of the NFκB signaling pathway to which drug resistant MM cells are suggested to be addicted [47]. By depleting MM cells of RelB and p52, they found significant downregulation of miR-221. They also showed that RelB-p52 complex repressed expression of pro-apoptotic gene BMF which is a known target of miR-221. These observations pinpoint induction of anti-apoptotic pathways as one potential mechanism of MM drug resistance by miR-221/222.

In another study, it was shown that expression of miR-21 was increased in MM cells following adhesion to BMSCs [8]. Treating U266 cells with miR-21 mimics rendered cells slightly resistant to Dex, Dox, and BTZ. The authors provide indirect evidence that the transcription factor NFκB may regulate miR-21 level. Inhibition of NFκB using the inhibitor BAY had inhibitory effect on miR-21 expression. This indicated that, at least in part, miR-21 expression in myeloma cells was regulated by NF-kB signaling, as also reported for miR-21 in a study on drug resistant B-cell lymphoma cell lines [35]. Furthermore, using in silico analysis by TFSEARCH v1.3 they found a NF-kB binding site in the 5′ flank region (∼470 bp from the transcription start site) of the miR-21 gene. Moreover, they identified RhoB as the direct target of miR-21. RhoB is a small GTPase member of the Rho family that is involved in integrin signaling and survival, among many other functions, however; it wasn't further explored in this study whether miR-21 functioned through integrin-mediated signaling following adhesion of MM cells to BMSCs.

Leone et al. [36] also showed that miR-21 targeted the tumor suppressor PTEN and interacted with AKT/ERK signaling pathways. Inhibition of miR-21 upregulated PTEN (and BTG, RhoB) in MM cells and abrogated AKT/ERK signaling suggesting that miR-21 could function through this oncogenic pathway. However, it isn't clear whether this would also happen in a drug-treated context to indicate the role of these pathways in miR-21-mediated drug resistance. In support of these findings, miR-21 was involved in resistance of B-cell lymphoma cells to cyclophosphamide, vincristine, adriamycin, and prednisone (CHOP) chemotherapeutic regimen through modulation of PI3K/AKT pathway and suppression of PTEN [35].

Importantly, miR-21 has been shown to contribute to oncogenic potential of STAT3 signaling pathway in MM cells [48]. In IL-6-dependent MM cells, IL-6 induced miR-21 in a strictly STAT3-dependent manner, ectopic expression of miR-21 in MM cells decreased their apoptosis in the absence of IL-6. Whether the same pathway will be modulated by miR-21 in a drug resistant MM model remains to be determined. Taken above findings as models, it seems plausible that oncomiRs in MM could promote cell cycle, proliferation or survival (anti-apoptotic) machinery of MM cells to prime them for chemoresistance while blocking their tumor suppressor targets.

The ways in which suppressed miRNAs may contribute to drug resistance in MM look further intricate and have not yet been clearly explored in any miRNA study in MM. The targets of these miRNAs could be oncogenes or anti-apoptotic proteins with basically high expression in MM. For instance, the well-known proto-oncogene and transcription factor c-MYC plays critical roles in MM pathogenesis and progression and is a strong driver of oncogenesis in lymphoma and MM models [49-53]. c-MYC has been shown to be involved in drug resistance in MM [54] and other hematologic malignancies such as acute myeloid leukemia (AML) [55, 56]. Furthermore, inhibition of c-MYC triggered apoptosis in MM cells [50]. While c-MYC-repressed miRNAs (miR-15a/16-1, miR-26a, miR-29, miR-34a, and miR-150) and their role in regulation of c-MYC-mediated responses (survival, inhibition of apoptosis) have been well characterized in c-MYC-driven lymphomas [57], how/whether miRNAs can regulate c-MYC-mediated functional responses such as drug resistance in MM is still not clear. It may be conceivable that overexpression of c-MYC builds the basis for chemoresistance, as it is a strong promoter of cell proliferation. Intriguingly, c-MYC induces drug resistance of AML blast cells by inhibition of *cell differentiation* [56]. On the contrary, in MM where tumor cells are terminally differentiated it may interact with other targets such as MCL-1 or survivin (a member of inhibitors of apoptosis (IAP) family) to induce drug resistance. This speculation is based on the observations that c-MYC induces resistance to Ara-C, dexamethasone and L-Asp in AML cell lines by upregulating survivin [58] and directs transcription of MCL-1 to control drug response of gastric cancer cells [59]. Although these functions of c-MYC have not been investigated in relation with miRNAs in MM, our recently published work shows that c-MYC regulates drug (PRIMA-1^Met^) response in MM cells by interacting with miR-29a (a tumor suppressor miRNA) [60]. We found that PRIMA-1^Met^ downregulated c-MYC and MCL-1 but upregulated miR-29a in MM cells and induced apoptotic cell death. c-MYC was identified as a direct target of miR-29a, upregulation of miR-29a suppressed c-MYC and inhibited MM cell proliferation. These findings led us to suggest that c-MYC might control expression of MCL-1, therefore inhibition of proliferation occurred due to c-MYC downregulation and apoptosis was induced due to MCL-1 inhibition by c-MYC [61]. Further investigation into whether this network could also control drug resistance in MM drug resistant models is ongoing. We have also yielded some preliminary findings that miR-137/197 could be involved in resistance of MM cells to BTZ by targeting MCL-1 [39].

**Figure 2:**
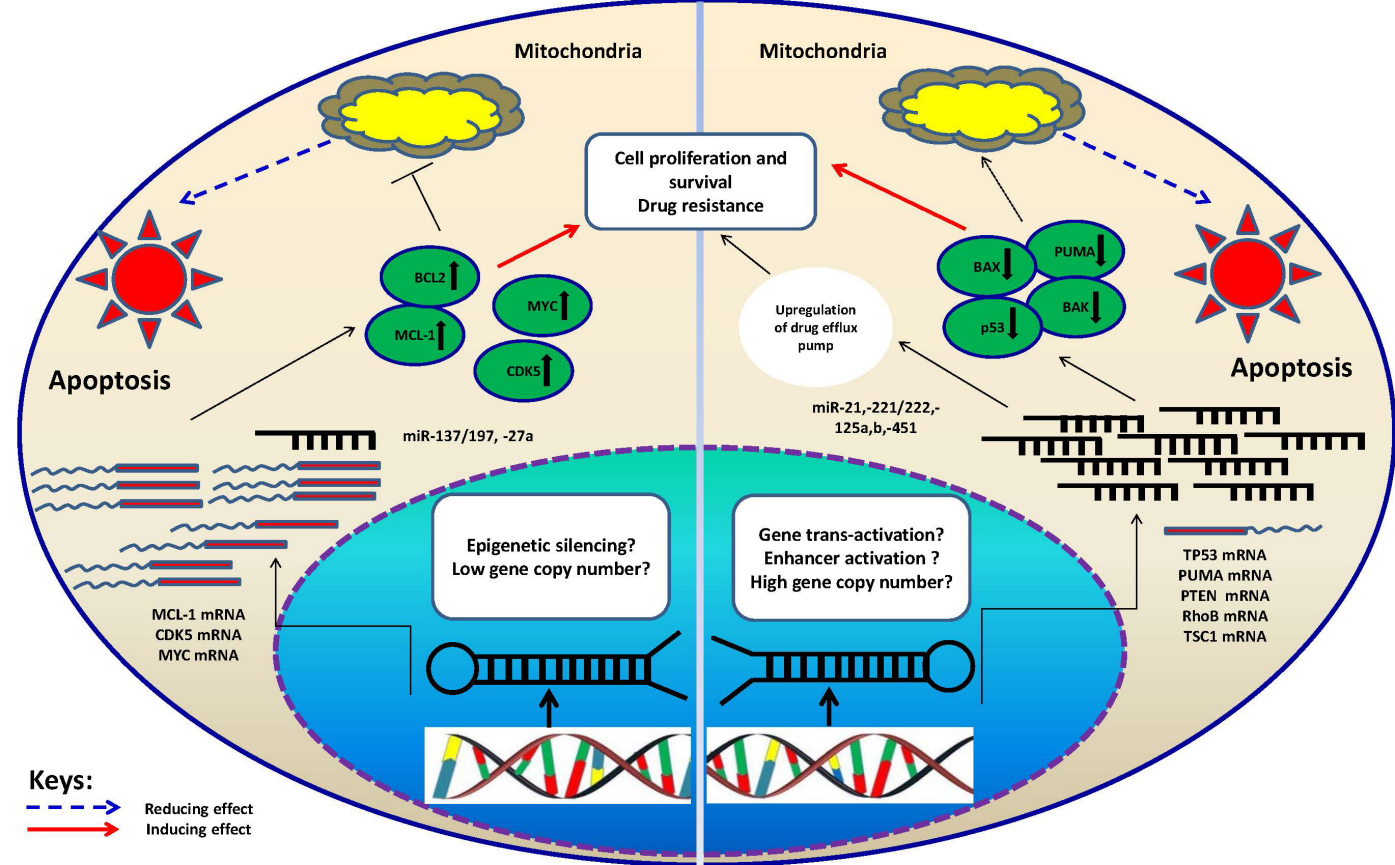
Schematic illustration of proposed mechanisms which may control miRNA-mediated drug resistance in MM The two categories of miRNAs, oncomiRs and tumor suppressors have been described. In case of oncomiRs (miR-21,-221/222,-125a/b,-451), which are overexpressed in MM cells, the targets will be downregulated. The latter are basically tumor suppressor or pro-apoptotic genes. This can lead to decline in drug-induced apoptosis hence drug resistance. On the contrary, for tumor suppressor miRNAs (miR-137/197, -27a) which are underexpressed in MM cells, the targets (oncogenes or anti-apoptotic proteins) will be upregulated leading to increase in cell proliferation and induction of drug resistance. It is important to note that mechanisms underlying alteration of above categories of miRNAs are not clearly understood, however; some clues suggest involvement of epigenetic silencing (tumor suppressors) and increase in gene copy number (oncomiRs). Whatever the mechanism, the inhibitory effect of miRNAs on their targets will be compromised in the oncogenic context contributing to optimization of conditions for tumor cells (resistance to drugs).

Among c-MYC-repressed miRNAs, tumor suppressor miR-15a/16-1 were first reported to be suppressed in chronic lymphoid leukemia (CLL) and found to target the anti-apoptotic protein BCL-2 [62]. These miRNAs, now known as regulators of cell cycle which also target CCND1, CCND2, CD25A, MCL-1 and WNT3 [63, 64], have been found downregulated in MM [65, 66] and shown to be involved in BMSCs-mediated drug resistance through induction of IL-6 and cell cycle progression [67, 68]. Finally, tumor suppressor miR-27a which was also reported to be downregulated in BTZ resistant MM cells and directly targeted CDK5 [29], could contribute to MM drug resistance by promoting cell cycle and proliferation, however; this miRNA can target P glycoprotein as well to induce multiple drug resistance in leukemic cells [69]. Taken together, tumor suppressor miRNAs may play role in MM drug resistance also by promoting cell cycle, proliferation or survival (anti-apoptosis) (illustrations at Figure 2).

Regarding the findings that BTZ could downregulate miR-21 [8] or miR-27a [29] in MM cells, involvement of at least these miRNAs in MM drug resistance sounds counterintuitive as it may be expected that the drug should not modulate the miRNAs. However; these studies did not utilize an appropriate model of *in vitro* drug resistance of MM cells nor did they use primary MM cells from MM patients resistant to drugs or at relapse, which would give more relevant indication of miRNA contribution to MM drug resistance.

Whether development of drug resistance might culminate in gain or loss of DNA hence over- or under-expression of miRNAs in MM cells is not known, however; increase in DNA copy number has been suggested to explain high expression of miR-221/222 in MM1R (dexamethasone resistant) relative to MM1S (dexamethasone sensitive) cells [9]. Chromosomal deletions harboring miRNA genes could also give evidence for downregulation of some miRNAs involved in MM drug resistance including miR-15a/16-1 cluster at 13q14.1 region which is frequently deleted in CLL and in more than 50% of MM patients [70, 71]. Interestingly, our group identified two tumor suppressor miRNAs, miR-137/197, to be downregulated in MM cells and mapped to a deleted region at chromosome 1 (1q21-22). However; epigenetic mechanisms especially DNA methylation at promoter CpG sites will also play an important role in regulation of expression of miRNAs as evidenced for several miRNAs in MM [72]. For example, epigenetic silencing of miR-137 at promoter CpG sites would be another mechanism underlying low expression of this miRNA in MM (unpublished data).

## MIRNAS, DRUG RESISTANCE AND THE CONCEPT OF MYELOMA CANCER STEM CELL

Identification of the clonal root of MM as supposedly the major source of drug resistance continues to be the challenging research area in MM. The investigations so far have shown that MM clones most probably originate from a ‘lymphocyte with memory B cell-like phenotype' outside of the bone marrow presumably germinal center of secondary lymphoid organs [73, 74]. This has generated a wide interest in elucidation of factors and mechanisms controlling “stemness” of these precursor cells giving rise to MM drug resistant malignant clones inside the bone marrow and also in targeting these clones [75]. Technically, MM cancer stem cells (CSCs) have been identified as a “CD138- side population (SP)” in flow cytometric analysis of MM cell lines and MM primary cells, with the most important stem cell-related markers and pathways including ALDH (aldehyde dehydrogenase), Hedgehog, Wnt, Notch and PI3K/Akt/mTOR being activated in MM SP cells [43, 76]. Furthermore, upregulation of drug efflux pumps (ABC transporters) in MM SP cells suggests that MM SP cells (CSCs) contribute largely to MM clones maintenance and (multi-) drug resistance [43, 76].

Surprisingly, miRNAs have recently been reported to be involved as critical regulatory elements in self-renewal, differentiation and proliferation of both hematopoietic stem cells (HSCs) and leukemia stem cells (LSCs) [77]. For instance, miR-125a and miR-125b (with known oncogenic roles and possible link to drug resistance in MM [41, 42] were shown to enhance self-renewal capability of HSCs and LSCs and induce AML in mouse models. Knowledge of the role of miRNAs in MM cancer stem cell (CSC) biology and particularly drug resistance is quite limited, however; Du et al. [43] in a pioneering study investigated the miRNA signature of MM side population (SP) cells which are believed to harbour the MM cancer initiating cells. They found the oncogenic miR-451 upregulated in SP cells; inhibition of miR-451 increased the effectiveness of BTZ, As_2_O_3_ and melphalan in SP cells by triggering apoptosis and decreasing clonogenicity, although latter cells exhibited some level of resistance to above agents compared to main population (MP) cells. This observation may at least partly explain relapse and refractoriness in MM patients. Moreover they demonstrated that miR-451 activated the PI3K/Akt/mTOR pathway in SP cells through its target tuberous sclerosis 1 (TSC1), this pathway is a well-known oncogenic pathway in cancers including MM [78-80]. Their findings give new insights into the regulatory role of miRNAs in MM SP cells (hence MM CSCs) biology and provide a promising approach to overcome drug resistance in MM. In contrast, miR-451 has been shown to be suppressed in CSCs of some other tumors indicating context-specific function of this miRNA [81]. Taken together, miRNA role in the regulation of MM CSC functional responses especially drug response will offer novel framework to elucidate more potential mechanisms underlying MM drug resistance and to provide more efficient tools to overcome this complication.

## APPROACHES TO EXPLOIT MIRNAS TO OVERCOME DRUG RESISTANCE IN MM

In light of the fact that miRNAs can target multiple genes involved in cancer promotion or repression, current era is witnessing the exploitation of miRNAs as new tumor cell targets/anticancer agents. The common sense in this approach is “inhibiting” the oncogenic miRNAs by injecting anti-oncomiRs and “restoring” the lost tumor suppressor miRNAs by injecting miRNA mimics. However; our knowledge on this respect to date has been obtained from pre-clinical evaluation of miRNAs in a variety of tumor xenograft models [12, 82]. Although in none of the MM-related studies miRNA therapeutic approach has been explored in a specific *in vivo* MM drug resistance platform, anti-oncomiRs or miRNA mimics have successfully proven to bear anti-tumor activity and display synergistic effects with anti-myeloma drugs [7, 9, 10, 36]. Of note, our recently published papers have also demonstrated pre-clinical anti-myeloma activity of miR-29a, -137, -197 mimics in MM xenograft models using novel lipid-based oligo delivery system (Neutral Lipid Emulsion) [39, 60]. It is important to note that although the studies discussed here suggest miRNAs as promising therapeutic tools in MM, no study so far has been conducted to apply these molecules to MM patients' clinical trials. Therapeutic potential, methodological issues (profiling, targeting) and challenges associated with efficient miRNA delivery have been reviewed elsewhere [83-85].

To study the clinical impact of miRNAs (either anti-oncomiRs or mimics) on MM drug resistance, *in vitro* findings on “drug resistant MM cell lines” need to be translated into mouse xenograft models. This means that the animal models should either provide a drug resistant phenotype of MM or harbour drug resistant MM cell line xenografts. Establishment of the former may require complicated genetic manipulations and seems far from feasibility, but for the latter xenografts can be made using drug resistant MM cell lines such as 8226-R5 as explained before [86]. Then the miRNA mimics or anti-oncomiRs should be injected with or without anti-myeloma drugs to explore the effects on tumor growth and expansion. It is important to note that generating a mouse model with humanized bone marrow microenvironment in these settings will yield more concrete results on the effects of miRNAs on MM drug resistance. The latter scenario will also address the unavoidable protective influence of bone marrow microenvironment components (BMSCs and extracellular matrix protiens) on MM cells.

## CONCLUDING REMARKS AND FUTURE PROSPECTS

The rising number of studies on miRNAs role in MM pathobiology, progression and prognosis and their therapeutic has created a new horizon in MM treatment especially in combination with efficient anti-myeloma drugs. However; drug resistance in MM continues to be the most challenging obstacle to MM therapy directing nowadays most research focus toward miRNAs to decipher the potential molecular mechanisms underlying resistance to therapy, however; investigation in this respect is still in its infancy in MM.

It should also be noted that drug resistance occurring due to adhesion of MM cells to BMSCs inside the bone marrow is now evidenced to be the major hub to focus in MM pathophysiology [87] hence even demanding far deeper and broader exploration into molecular mechanisms of MM drug resistance. We have found that BMSCs suppress several miRNAs including miR-101-3p and upregulate the oncogene c-FOS and the anti-apoptotic protein survivin in MM cells following cell-cell interaction even when the system is exposed to BTZ. These findings imply involvement of miR-101-3p in BMSCs-induced resistance to BTZ probably through c-FOS or survivin.

As the last remark, given involvement of miRNAs in drug resistance in MM, some important questions concerning the experimental models used will be raised: How are miRNAs in drug resistant cells suppressed or upregulated? Are these changes actually happening due to the oncogenic process in the first place paving the way for development of drug resistance? Are these by-products of the neoplastic phenomenon? Or, they arise following gain or loss of the genetic material due to drug treatment? Perhaps until finding appropriate answers to these questions, one need to be cautious to justify actual miRNA involvement in MM drug resistance inside the MM patient's bone marrow microenvironment based on *in vitro* and *in vivo* observations. Furthermore, in spite of applicability of strategies in *in vivo* studies, the major issue is still with the ways miRNAs should be applied to human subjects, i.e. directing the miRNAs to the bone marrow microenvironment and targeting only the MM clones.
